# Effect of Center Determination Method, Point Cloud Filtering and Cross-Section Type on the Accuracy of Tree Stem Volume Estimation Using Laser Scanning

**DOI:** 10.3390/s26144405

**Published:** 2026-07-11

**Authors:** Patrik Kúdela, Matej Ján Dominka

**Affiliations:** National Forest Center, Science and Research Division, Department of Forest Policy, Forest and Game Management, T. G. Masaryka 2175/22, 960 01 Zvolen, Slovakia; matej.dominka@nlcsk.org

**Keywords:** computed tomography, laser scanning, point cloud, volume calculation

## Abstract

Static laser scanning is an advanced approach to timber volume estimation whose accuracy now matches or exceeds that of traditional forestry methods for volume estimation, enabling its practical application in woodworking management. This study evaluates how stem volume estimation accuracy from point clouds is affected by stem surface condition (with and without bark), cross-sectional area calculation methods, point cloud filtering, and stem center determination. Results show that the stem surface condition fundamentally controls the direction and magnitude of systematic errors. For stems with bark, the cross-sectional area derived from the squared diameter caused systematic volume underestimation, which increased after filtering, whereas the quadratic mean diameter led to overestimation that was substantially reduced by filtering and approached reference values obtained from computed tomography. For debarked stems, the squared diameter method produced systematic overestimation that was effectively mitigated by filtering, while the quadratic mean diameter resulted in persistent overestimation. The volumetric method itself had only a secondary influence on accuracy. Volume estimation accuracy was primarily governed by cross-sectional area formulation and stem surface condition. For logs with bark, the squared arithmetic mean radius produced systematic volume underestimation that increased by approximately 1% after filtering, whereas the quadratic mean radius overestimated volume by about 2% after filtering. For debarked logs, volume overestimation decreased from 4.05–4.72% to 1.57–1.80% after filtering when using the squared arithmetic mean radius, while overestimation remained high (6.55–6.92%) when the quadratic mean radius was applied. In contrast, the choice of volumetric method and center determination affected volume estimates by less than 1%. These findings confirm the high potential of static laser scanning and emphasize the need for point cloud-adapted computational models.

## 1. Introduction

Forest ecosystems play a fundamental role in providing natural resources and ecosystem services, which makes them essential for biodiversity conservation, soil protection, and climate change mitigation [1,2,3]. Accurate assessment of forest stand characteristics is therefore a key prerequisite with significant implications for forest management planning, forest fire modeling, and carbon stock estimation [3,4,5].

Methods for measuring the structural characteristics of forest stands have undergone rapid development, evolving from conventional field-based approaches to modern technology-driven techniques. Traditional measurement methods are capable of providing direct and highly reliable data; however, they are associated with substantial financial costs and high labor demands. Consequently, the need to acquire data rapidly and at minimal cost has given modern measurement approaches a critical advantage [6]. Although contemporary technologies significantly reduce the time required for field data collection, the acquired datasets require extensive post-processing to ensure their usability and relevance for end users.

Recent advances in terrestrial laser scanning (TLS) technologies and computed tomography (CT), in combination with mathematical and statistical dendrometric methods, enable relatively precise quantification of wood volume [7,8,9,10]. Among currently available techniques, TLS represents one of the most accurate methods for acquiring forestry-relevant data [11]. Until recently, laser-based technologies were primarily used for applications such as mapping [12,13], imaging [11,14], and surveying [15]. At present, TLS is considered the most advanced approach for obtaining high-resolution information directly from forest environments [15]. TLS systems are predominantly applied at the local scale, where they enable the capture of highly detailed geometric representations of objects, including individual trees and logs [11]. However, TLS has its own limits, hindering its applicability in the field. TLS is limited mainly by occlusion, error sources, and difficult workflows, with added constraints from cost, processing burden, and incomplete standards [16,17]. TLS accuracy depends strongly on scan geometry, range, atmosphere, instrument condition, and the scanned surface, so precision in one setting does not transfer automatically to another [18,19]. In dense forest conditions, TLS struggles mainly with occlusion, incomplete sampling, and noisy point clouds in terrestrial laser scanning, which reduce detection of stems, branches, and upper-canopy structure and can bias forest metrics [20,21,22]. These problems are strongest in multilayer stands with an understory, small stems, dense crowns, steep terrain, or leaf-on and windy conditions, although multi-scan designs and combined ground-plus-above-canopy measurements can reduce them substantially [23,24,25].

CT is a non-invasive and non-destructive imaging technology originally developed for medical diagnostics [26]. Over time, CT has been progressively adopted in soil sciences [27] and plant sciences [28], with its first application in wood science reported by [29]. CT systems enable the analysis of entire tree stems and are particularly suitable for investigating internal qualitative properties of wood that are not observable on the stem surface. Despite these advantages, the practical application of CT in forestry and wood science remains limited due to the restricted availability of suitable devices and their high acquisition costs [10,30,31].

The whole tree stem shape cannot be modeled conveniently by a single classical geometry. The average tree stem form is modeled as a truncated neiloid at the lowest part, a truncated paraboloid at the central section and a paraboloid or cone at the top [32,33,34]. The main practical problem is that it is not possible to know exactly where one geometry ends and another begins [35].

A similar issue applies to individual logs, which rarely exhibit perfectly straight and regular geometry, but instead occur in a wide range of shapes and taper forms. Consequently, traditional forestry methods for log measurement are inherently prone to estimation errors [36]. Although CT scanners currently represent the most accurate measurement technology, their acquisition costs are prohibitively high for many small- and medium-sized sawmills. Static laser scanning systems may therefore provide a practical and economically feasible alternative.

The objective of this study was to evaluate the influence of two different approaches for deriving the center of point clouds from laser scanning data, as well as two simplified approaches for calculating the log cross-sectional area, the square of the arithmetic mean radius and the quadratic mean radius, on the accuracy of roundwood volume estimation. Furthermore, this study compared two volume calculation approaches, the Huber method and the Frustum method, in terms of their mutual differences and their accuracy relative to a reference volume obtained from CT scanning. In addition, the effect of point cloud smoothing using the Open3D library implemented in the Python programming language was assessed with respect to its impact on volume estimation precision.

## 2. Materials and Methods

### 2.1. Target Tree Species

**Figure 1:**
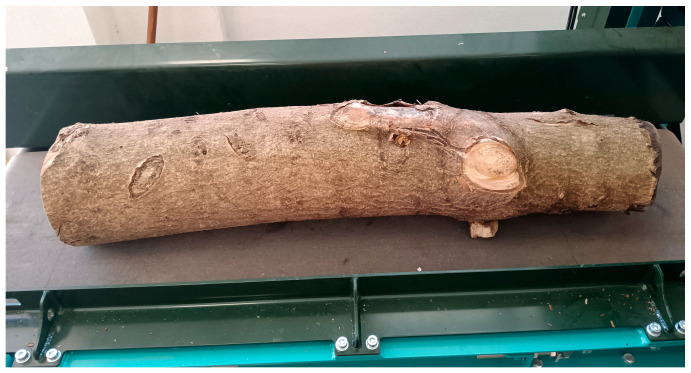
Log on the conveyor belt.

For the analysis, log data with and without bark stored in a timber yard were selected. In total, 13 logs representing six tree species were included in the study. The dataset comprised five logs of *Paulownia* spp. (four with bark and one without bark), three logs of *Quercus* spp. L. (one with bark and two without bark), two logs of *Carpinus betulus* L. (one with bark and one without bark), one log of *Picea abies* (L.) H. Karst. with bark, one log of *Pinus sylvestris* L. without bark, and one log of *Abies alba* Mill. without bark. The average log length was approximately 1 m (as shown in Figure 1).

### 2.2. Scan Data Acquisition

**Figure 2:**
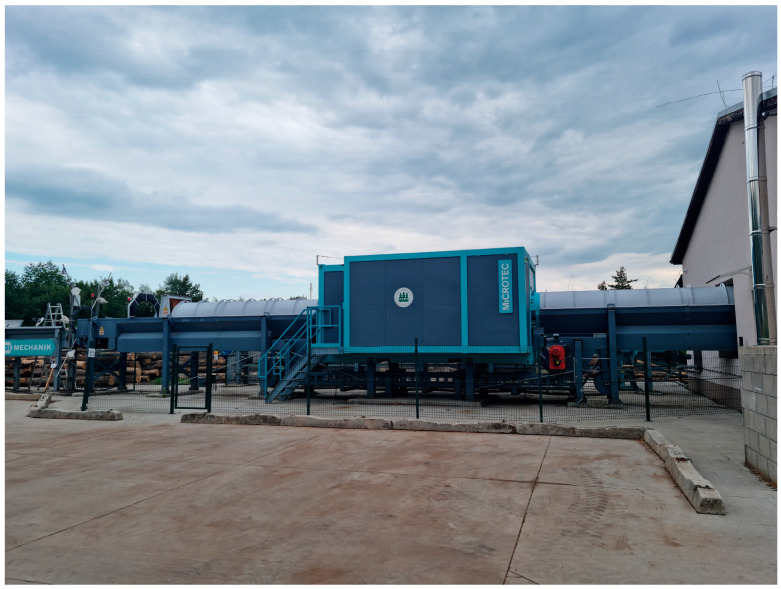
Three-dimensional CT scanner manufactured by Microtec based in Italy.

The logs were scanned using a three-dimensional CT scanner manufactured by Microtec based in Italy (as shown in Figure 2). During scanning, each log was conveyed through the scanning device and exposed to X-ray radiation. Differences in X-ray absorption by individual wood components enable the determination of their density. Based on tomographic inversion, the reconstruction algorithm generates a density image for each scanned cross-sectional slice, resulting in a three-dimensional model of the log. The spatial resolution of the CT scanner was limited to 10 mm in the longitudinal direction and 2 × 2 mm in the transverse direction. The grayscale pixel intensity, referred to as the CT image digital number (DN), represents the attenuation of X-ray radiation, which depends on the amount of absorbed energy. Materials with lower density are displayed as darker gray tones in CT images, whereas materials with higher density appear as lighter gray tones. Wood density within cross-sections exhibits pronounced spatial variability, driven by annual ring structure and the presence of qualitative features such as knots, cracks, or decay [37].

The output of the CT scanning process consists of a series of TIFF images representing individual cross-sections of the log at 10 mm intervals. Thus, scanning a log with a length of 1000 mm produces a dataset of 100 cross-sectional slices. These slices were subsequently assembled into a three-dimensional log model. The sequence of cross-sections was imported into the specialized software 3D Slicer (version 5.8.0) [38,39]. Each slice was assigned a thickness of 10 mm, and a volumetric rendering function was used to generate the 3D log model. The resulting model was composed of voxels with dimensions of 2 × 2 × 10 mm. The volume of the 3D log model was calculated using the statistical segmentation function, which determines volume based on the number and size of voxels [39]. The resulting log volume was used as a reference value against which the selected log volume estimation methods were evaluated.

**Figure 3:**
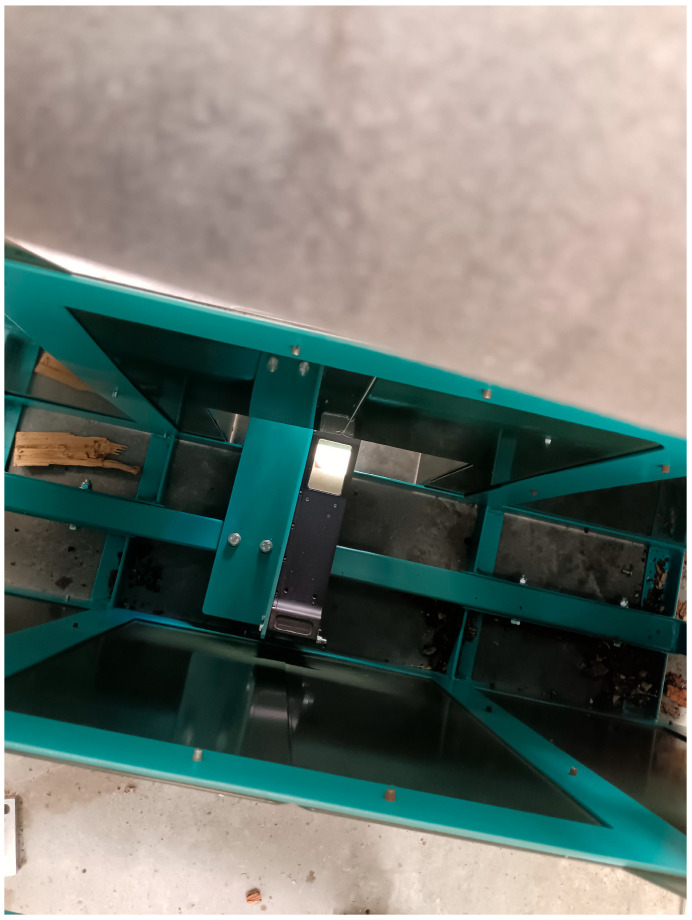
Gocator Laser Profiler (model 2670).

**Figure 4:**
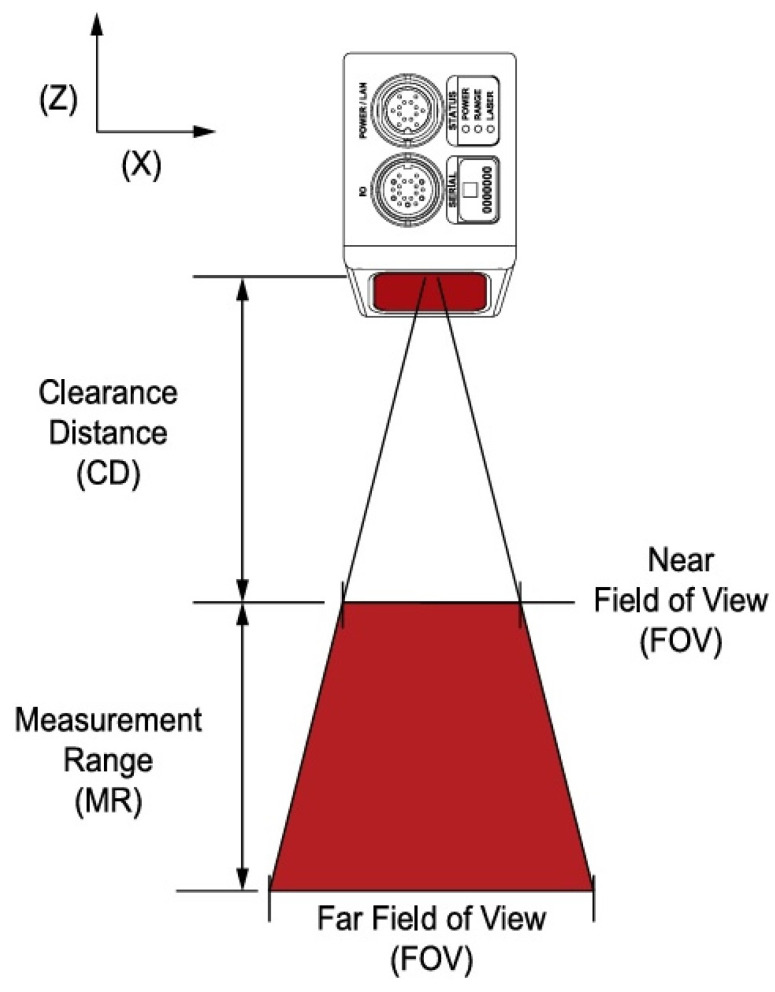
Trapezoidal field of vision of Gocator Laser Profiler (model 2670).

In the subsequent step, each log was scanned using a Gocator Laser Profiler (model 2670), consisting of three sensors that simultaneously captured the surface geometry of the log (as shown in Figure 3). The field of vision of the laser has a trapezoidal shape. The distance between points is closer at the near range than at the far range, see Figure 4.

**Figure 5:**
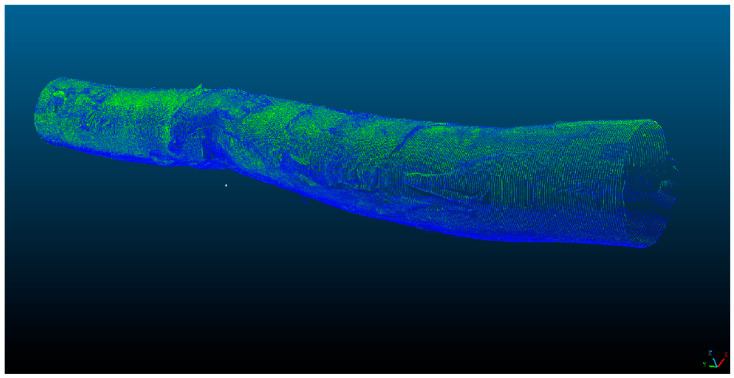
Point cloud representation of the log.

The scanning process produced separate point clouds generated by each sensor with a spatial resolution of 1 mm. Each point in the cloud represents a voxel with a volume of 1 mm^3^. The point clouds obtained from the individual sensors were subsequently merged into a single unified point cloud (see Figure 5), from which a three-dimensional model of the log was constructed. The volume of the resulting 3D log model was calculated within the Python programming environment.

### 2.3. Data Post-Processing

The center of the acquired point cloud was determined using two different approaches. The first approach relied on the direct center provided by the scanning device, which is defined by the intersection of the directional beams of the three laser sensors. The second approach involved a computational method based on Principal Component Analysis (PCA). In the PCA-based approach, the mean coordinates of the points were calculated for each cross-sectional slice of the log, yielding the centroid of the data. Subsequently, all points were translated in space so that the centroid was shifted to the origin. The coordinates of the centroid were subtracted from the coordinates of each point, and a covariance matrix was constructed from the centered data.

To ensure the symmetry of the covariance matrix, spectral decomposition was performed, resulting in the computation of eigenvalues and their corresponding eigenvectors. The largest eigenvalue and its associated eigenvector defined the first principal component of the PCA, representing the direction of maximum variance in the data. This eigenvector was subsequently normalized to unit length, yielding a unit direction vector that defined the orientation of the point cloud axis in three-dimensional space. For subsequent calculations, it was necessary to determine the cross-sectional area of the log. Because the point cloud within each cross-section, bounded by its envelope curve, formed a geometrically complex and irregular shape, two simplified approaches for cross-sectional area estimation were applied. The first approach is based on the square of the arithmetic mean radius and is defined as:

(1) $$S=\pi {\left(E[r]\right)}^{2}$$

The second approach employs the quadratic mean radius, with the cross-sectional area calculated as:

(2) $$S=\pi \;E[r^{2}]$$

where r represents the distance of individual point cloud points from the log axis, and E[⋅] denotes the expected (mean) value.

The resulting data were subsequently filtered to remove statistical outliers and noise using the Python programming environment and the Open3D library, which is specifically designed for point cloud processing and filtering. To compare the accuracy of log volume estimation, four additional calculation methods were applied, namely the Huber method with two variants and the Frustum method, also with two variants. The point cloud of each log was divided into transverse slices with a thickness of 2 cm. These slices served as the basic units for volume computation using the individual methods. The total log volume was subsequently determined as the sum of volumes of all slices.

**Figure 6:**
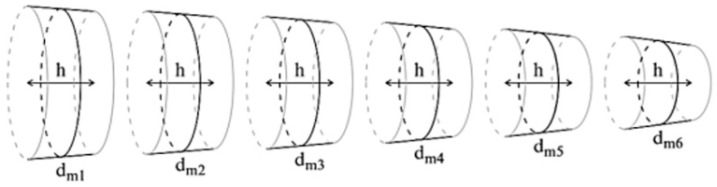
Tree data measurement with Huber’s formula [40].

The Huber method is a well-established method in forestry (see Figure 6) [36,40]. It is used to calculate volume based on the cross-sectional area at the midpoint. The method was implemented through two variants.

Each transverse slice was represented by its own point cloud with a predefined center. For each slice, the distance from the center to every point in the two-dimensional plane was computed, and the mean radius $\bar{r}$ was derived from these distances. The cross-sectional area of the circular slice was calculated using two alternative formulations:

(3) $$A_{1}=\pi ({\bar{r})}^{2}$$

(4) $$A_{2}=\pi \;\bar{r^{2}}$$

where π=3.14, $\bar{r}$ denotes the arithmetic mean of the distances of the points from the slice center, and $\bar{r^{2}}$ represents the mean of squared distances.

The volume of an individual slice was then calculated as:

(5) $$V_{i}=A_{i}\cdot L$$

where $A_{i}$ is the cross-sectional area computed using one of the above formulations, and L is the slice length in centimeters.

The total log volume was obtained as the sum of the volumes of all slices:

(6) $$V=\sum_{i=1}^{n}V_{i}$$

where $V_{i}$ represents the volume of the i-th slice.

The Frustum method represents an exact geometric case of Newton’s method for volume calculation. The Frustum formula assumes linear taper, whereby the midpoint radius is equal to the arithmetic mean of the end radii. In contrast, Newton’s method approximates volume using an independently measured midpoint cross-sectional area.

Frustum was likewise implemented in two variants. Each transverse slice was represented by a point cloud with a defined center. At both the beginning and the end of each slice, the distances of points from the center were computed in the two-dimensional plane, and the corresponding mean radii were determined as in the Huber method.

The volume of an individual slice was calculated using the Frustum formula:

(7) $$V_{i}=\frac{L}{3}\left(A_{i1}+A_{i2}+\sqrt{A_{i1}\cdot A_{i2}}\right)$$

where $A_{i1}$ and $A_{i2}$ are the cross-sectional areas at the beginning and end of the slice, computed using the same formulation, and L is the slice length in centimeters.

The total log volume was determined as the sum of the volumes of all slices:

(8) $$V=\sum_{i=1}^{n}V_{i}$$

where $V_{i}$ denotes the volume of the individual slices.

The same procedure was used on the data after the application of Open3D library. From the Open3D library we used the method for removing the statistical outliers on the point cloud data called “remove_statistical_outlier”. Statistical outlier removal was performed using 30 neighboring points and a standard deviation ratio of 2. The selected parameters were based on commonly applied point cloud denoising practices and were empirically verified to effectively suppress isolated noise while preserving the geometric characteristics of the scanned stem [41,42].

## 3. Results

**Figure 7:**
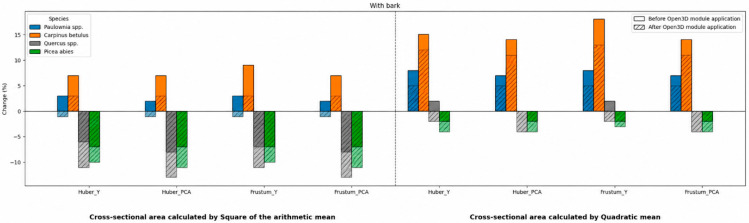
Comparison of volume estimates obtained from laser scanning with the reference CT-based volume for logs with bark.

Figure 7 illustrates the comparison of log volumes estimated using the Huber method and the Frustum method against the reference volume obtained from CT scanning for logs with bark.

In the first half of the figure, volumes are calculated using cross-sectional areas derived from the square of the arithmetic mean radius, whereas the second half presents results based on cross-sectional areas computed using the quadratic mean radius. Solid bars represent volume estimates derived from the original point cloud data obtained directly from the laser scanner. Hatched bars indicate volume estimates after statistical outlier removal and surface smoothing performed using the Open3D library in the Python programming environment. The computations were carried out on a workstation equipped with an Intel^®^ Xeon^®^ CPU E5-2665 @ 2.40 GHz, 32 GB RAM, and an NVIDIA Quadro K2200 GPU with 4 GB memory.

The application of Open3D systematically amplifies volume underestimation when the cross-sectional area is computed using the square of the arithmetic mean radius, increasing the negative bias by approximately 1% on average across all analyzed tree species and for both volume computation methods. In contrast, when the quadratic mean radius is used, the application of Open3D leads to a substantial reduction in volume overestimation, lowering the bias to approximately 2%, thereby bringing the results closer to the CT-based reference values.

Despite differences in the absolute magnitude of deviations among individual tree species, the sign of the systematic error remains consistent. The use of the square of the arithmetic mean radius consistently results in volume underestimation, whereas the quadratic mean radius leads to systematic overestimation. Neither the application of Open3D nor the choice of center determination method altered the relative ranking of volume estimates among the analyzed tree species.

The comparison between the Huber method and the Frustum method revealed only minor differences in the resulting volume estimates, typically within 1%. In contrast, the method used to compute the cross-sectional area represents the dominant factor affecting volume estimation accuracy, with differences between the two approaches ranging from 3% to 5%. Determining the log center using PCA resulted in a consistent reduction in the estimated volume by approximately 0.4%. This effect was stable across all tree species and was independent of the application of Open3D.

**Figure 8:**
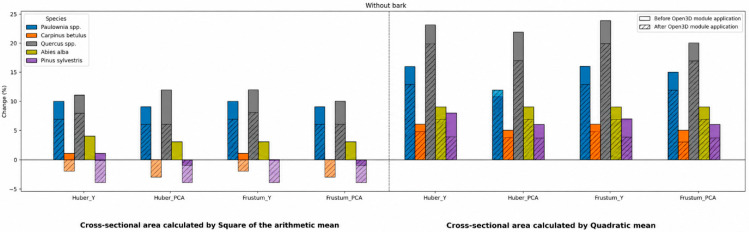
Comparison of volume estimates derived from laser scanning with the CT-based reference volume for logs without bark.

Figure 8 presents a comparison of volumes estimated using the Huber method and the Frustum method against the CT-derived reference volume for logs without bark. The first half of the figure shows results based on cross-sectional areas calculated using the square of the arithmetic mean radius, while the second half presents results obtained using the quadratic mean radius.

Prior to the application of the Open3D module, volume estimates computed using the square of the arithmetic mean radius exhibited a clear systematic overestimation relative to the CT-based reference volume, with mean deviations ranging from 4.05% to 4.72%. After point cloud filtering using Open3D, the overestimation was substantially reduced to approximately 1.57–1.80% consistently across all analyzed tree species. In contrast, the use of the quadratic mean radius resulted in a pronounced increase in volume overestimation, reaching 6.64–7.31% prior to the application of Open3D. In this case, the application of Open3D had only a limited corrective effect, as the overestimation decreased only marginally to approximately 6.55–6.92%.

The comparison between the Huber method and the Frustum method revealed only minor differences in the resulting volume estimates and proved to be of secondary importance in all cases, with discrepancies between the two approaches generally remaining below 1%. Determining the log center using PCA led to a consistent reduction in the estimated volume of approximately 0.3% compared to the center directly defined by the scanning device.

## 4. Discussion

The principal methodological contribution of this study is the identification of the relative importance of stem surface condition, cross-sectional area formulation, point cloud filtering, and center determination for TLS-based stem volume estimation. The results demonstrate that cross-sectional area formulation governs systematic bias, whereas filtering and center determination primarily act as corrective factors.

A comparison of the volume estimation results for logs with and without bark demonstrated that the presence of bark had a fundamental influence on the nature of systematic error in volume calculation. For logs with bark, the computation of cross-sectional area using the square of the arithmetic mean radius resulted in a systematic underestimation of volume, whereas for debarked logs the same computational approach led to a systematic overestimation. This behavior indicates differences in the distribution of radii within cross-sections depending on the presence of bark and its spatially variable thickness.

Among the compared methods, the Huber and Frustum approaches exhibited the smallest measurable effect on the final volume estimates. Because the logs were divided into 100 segments, the influence of geometric distortion was substantially attenuated. This observation is consistent with the findings of [36], who reported that when a log is divided into 30 or more segments, the error associated with the Huber method does not exceed 2.5%.

The determination of the point cloud center had a measurable effect on the final estimated volume. Numerous methods for estimating the center of a stem or log cross-section from point cloud data have been described in the literature, including circle-, ellipse-, cylinder-, polygon-, and spline-fitting approaches [40,43,44,45]. Among these, circle-fitting methods are the most commonly applied and include techniques based on the Hough transform, RANSAC, robust least-squares procedures, and algebraic or geometric solvers such as Kasa, Pratt, Taubin, Levenberg–Marquardt, and Gauss–Newton algorithms [46,47,48]. In the present study, we compared two fundamental approaches: defining the center directly from the scanner geometry and estimating it using PCA. Although more sophisticated methods may improve robustness when point clouds are noisy, incomplete, or affected by occlusion, the influence of center determination on volume estimation remained relatively small (approximately 0.3–0.4%) compared with the substantially larger effects of cross-sectional area formulation and stem surface condition. In this study, two fundamental approaches were compared: defining the center directly from the intersection of the scanner sensor beams, and estimating the center using PCA.

Center determination using PCA led to a consistent reduction in estimated volume of approximately 0.3–0.4% compared to the center defined by the scanner geometry, regardless of bark presence and independent of point cloud smoothing using the Open3D library. This effect can be attributed to a slight attenuation of local eccentricities by PCA, resulting in a reduced effective diameter during cross-sectional area computation. Although this difference was not statistically significant, its consistent direction across all analyzed scenarios indicates a small systematic bias. However, compared with the substantially larger effects of cross-sectional area formulation and stem surface condition, the practical influence of center determination on volume estimation is negligible. The consistently small magnitude of the observed differences suggests that defining the center directly from the scanner geometry provides a fast and sufficiently accurate solution for logs with regular geometry. Nevertheless, for logs exhibiting pronounced eccentricity, curvature, or irregular cross-sectional shapes, more advanced center determination methods may improve robustness and should be evaluated in future studies.

The computation of cross-sectional area, or more generally the determination of an appropriate envelope curve for scanned point data, has been the subject of sustained research interest [49,50,51]. Accurate definition of the envelope curve is critical for subsequent calculations across a wide range of applications. Ref. [52] demonstrated that estimating envelope curves (e.g., circles) from incomplete or noisy data constitutes a numerically and geometrically challenging problem. In response to these challenges, numerous methods for circular envelope approximation have been developed [53,54,55,56,57].

In this study, two simplified approaches were applied for estimating circular cross-sectional area: computation based on the square of the arithmetic mean radius and computation based on the quadratic mean radius. For both approaches, the Open3D library was additionally applied to filter the point cloud data, remove outliers, and smooth the surface geometry.

Point cloud filtering using Open3D exhibited a consistent corrective effect when cross-sectional area was computed using the square of the arithmetic mean radius, but with opposite directional outcomes depending on bark presence. For logs with bark, filtering deepened the systematic underestimation of volume, whereas for debarked logs it resulted in a pronounced reduction in overestimation and improved agreement with the CT-derived reference volume. When the quadratic mean radius was used, a systematic overestimation of volume was observed in both cases (with and without bark). While the application of Open3D substantially reduced this overestimation for logs with bark, the overestimation for debarked logs remained high even after point cloud filtering. This finding indicates the dominance of model error arising from the mathematical formulation of the quadratic mean radius itself, which amplifies the influence of extreme radius values and exhibits reduced robustness to geometric irregularities in cross-sectional shape. We assume that the application of Open3D may introduce a systematic bias toward volume underestimation due to the selected filtering parameters. Specifically, the statistical outlier removal procedure, which is based on the standard deviation of neighboring points, likely preferentially removes points located farther from the central axis of the log. Consequently, the reconstructed geometry may be reduced, leading to an underestimation of the calculated volume.

Within this study, the most promising combination for practical application was identified as the computation of cross-sectional area using the quadratic mean radius combined with point cloud filtering using the Open3D library. This approach yielded the smallest systematic deviation from the reference volume obtained by CT scanning and exhibited consistent behavior across all analyzed tree species. The LiDAR scanner acquires point cloud data from a trapezoidal viewing geometry, resulting in small positional deviations around the Z-axis. Consequently, the reconstructed point cloud contains a substantial number of outlying points. Under these conditions, the quadratic mean radius is disproportionately influenced by extreme radial values and is therefore more prone to overestimating the log volume (see Table 1).

The results clearly demonstrate that the accuracy of log volume estimation is far more strongly influenced by the method used to compute cross-sectional area than by the choice of the volumetric integration method itself. A comparison between logs with bark and debarked logs indicates that bark removal leads to an amplification of systematic volume overestimation, particularly when cross-sectional area is calculated using the quadratic mean radius, and simultaneously increases the importance of selecting an appropriate combination of computational procedures. It is assumed that the increased variability in the point cloud in debarked logs results in higher sensitivity of cross-sectional area estimation to local eccentricities, which subsequently manifests as systematic overestimation of the final volume.

Although the tested tree species exhibited consistent trends, the limited sample size and unequal species representation did not permit robust species-specific conclusions. Future studies should evaluate the proposed methodology using a larger dataset covering a broader range of species, stem dimensions, and bark characteristics.

Across Europe, numerous volume equations have been developed for the main European tree species [58,59], while in some countries the collection of empirical data and derivation of national volume equations have only been completed relatively recently [60]. The findings of this study suggest that systematic errors in the form of volume overestimation or underestimation are not primarily species-dependent, but rather arise from the mathematical formulation of the cross-sectional area computation. This insight allows a certain degree of generalization of the results across tree species. Nevertheless, the size of the current dataset, comprising six tree species, is insufficient for deriving robust volume equations that would be representative for individual species.

## 5. Conclusions

Static laser scanning represents a modern method for fast, precise and cheap measuring of wood volume at the sawmills. Accuracy of static laser scanning already surpasses that of traditional forestry approaches and enables its effective implementation in woodworking. The application of this method allows for cost reduction while simultaneously providing faster and more precise acquisition of wood volume data. The aim of this study was to analyze the influence of different log volume calculation procedures on the accuracy of results obtained from point clouds. Particular emphasis was placed on comparing the volume estimation of logs with and without bark, as well as evaluating the impact of the cross-sectional area computation method, point cloud filtering, and the determination of the log center.

The results indicated that the presence of bark had a significant effect on the behavior of systematic volume estimation errors. For logs with bark, computing the cross-sectional area using the arithmetic mean squared resulted in systematic underestimation of volume, which was further amplified after point cloud filtering using the Open3D library. In contrast, using the quadratic mean radius produced systematic overestimation of volume, which could be substantially reduced through Open3D filtering, bringing results closer to the reference values obtained from CT scanning. For debarked logs, the behavior of the calculation methods differed. The arithmetic mean squared method led to systematic overestimation of volume, which could be effectively reduced by point cloud filtering, achieving the lowest deviation among all analyzed combinations. However, using the quadratic mean radius in this case resulted in pronounced and persistent overestimation, which remained significant even after data filtering, indicating that model-inherent errors dominated over errors arising from input data quality.

The findings confirm the high accuracy of laser scanning for determining wood volume. Future research should focus on deriving new volume equations for selected forest tree species based on point clouds, enabling the development of algorithms for rapid volume estimation directly in the stand without additional data processing. The choice of volumetric integration method, whether Huber’s method or the Frustum method, had only a secondary effect on volume calculation accuracy. Instead, the accuracy of log volume estimation is primarily influenced by the cross-sectional area computation method and the surface condition of the log (with or without bark), whereas the choice of volumetric method and center determination approach are of complementary importance.

The results highlight the need for further research aimed at disentangling the effects of point cloud quality and computational model formulation on the accuracy of log volume estimation. Special attention should be given to a systematic comparison of different point cloud filtering techniques and their impact on the statistical properties of cross-sections, particularly considering the varying sensitivity of different mean types to extreme values.

Another important research direction is the quantification of the effects of point cloud density and spatial distribution on volume estimation accuracy. Experiments simulating degraded point cloud quality would allow the definition of the minimum point density and spatial homogeneity requirements needed to achieve a desired level of volume estimation accuracy. Such simulations could be implemented by systematically subsampling the original point clouds to progressively reduce point density, introducing artificial occlusions to mimic shadowing effects encountered during terrestrial laser scanning, or adding controlled positional noise to simulate measurement uncertainty. The resulting volume estimates could then be compared with the reference values to determine the threshold conditions under which estimation accuracy begins to deteriorate.

From the perspective of the computational model, investigating alternative or adaptive formulations for cross-sectional area that account for local geometric variability in the log appears promising. Such approaches could reduce systematic error without the need for empirical corrections. Further research should also focus on a more detailed evaluation of the effect of log axis and center determination, since even small systematic offsets may accumulate in large datasets. Finally, the use of statistical modeling of systematic errors represents a prospective avenue, enabling not only the post hoc correction of results but also the quantification of uncertainty in volume estimation.

## Figures and Tables

**Table 1:** Decision matrix of methodological recommendations.

Used Method	Bark	Calculation of Area of Slice	Open3D	Error Estimation	Recommendation
Standard scan	With	Quadr. mean	No	Slight underestimation	No
Standard scan	With	Quadr. mean	Yes	Significant underestimation	No
More precise calculation of volume	With	Square of the mean	Yes	Slight overestimation	Yes
More precise calculation of volume	With	Square of the mean	No	Significant overestimation	No
Standard scan	Without	Quadr. mean	No	Significant overestimation	No
Standard scan	Without	Quadr. mean	No	Low deviation	Yes
More precise calculation of volume	Without	Square of the mean	Yes/No	Significant overestimation	No

## Data Availability

Data are available on this URL: https://kaggle.com/datasets/d321870d83954f39d48ee69ebb198db457276389c121b5c5adedf1ff6fe1184c (accessed on 4 July 2026).
